# Presynaptic GABAergic inhibition regulated by BDNF contributes to neuropathic pain induction

**DOI:** 10.1038/ncomms6331

**Published:** 2014-10-30

**Authors:** Jeremy Tsung-chieh Chen, Da Guo, Dario Campanelli, Flavia Frattini, Florian Mayer, Luming Zhou, Rohini Kuner, Paul A. Heppenstall, Marlies Knipper, Jing Hu

**Affiliations:** 1 Centre for Integrative Neuroscience, Otfried-Mueller-Strasse 25, 72076 Tübingen, Germany; 2 Hearing Research Centre, Elfriede Aulhornstrasse 5, 72076 Tübingen, Germany; 3 Laboratory for NeuroRegeneration and Repair, Center for Neurology, Hertie Institute for Clinical Brain Research, 72076 Tübingen, Germany; 4 Pharmacology Institute, University of Heidelberg, Im Neuenheimer Feld 584, 69120 Heidelberg, Germany; 5 Mouse Biology Unit, European Molecular Biology Laboratory (EMBL), Via Ramarini 32, 00016 Monterotondo, Italy

## Abstract

The gate control theory proposes the importance of both pre- and post-synaptic inhibition in processing pain signal in the spinal cord. However, although postsynaptic disinhibition caused by brain-derived neurotrophic factor (BDNF) has been proved as a crucial mechanism underlying neuropathic pain, the function of presynaptic inhibition in acute and neuropathic pain remains elusive. Here we show that a transient shift in the reversal potential (*E*_GABA_) together with a decline in the conductance of presynaptic GABA_A_ receptor result in a reduction of presynaptic inhibition after nerve injury. BDNF mimics, whereas blockade of BDNF signalling reverses, the alteration in GABA_A_ receptor function and the neuropathic pain syndrome. Finally, genetic disruption of presynaptic inhibition leads to spontaneous development of behavioural hypersensitivity, which cannot be further sensitized by nerve lesions or BDNF. Our results reveal a novel effect of BDNF on presynaptic GABAergic inhibition after nerve injury and may represent new strategy for treating neuropathic pain.

## Abstract (web summary)

Disinhibition of neural activity in the spinal cord is implicated in neuropathic pain. Chen *et al.* show that disinhibition of neural activity arises from a shift in reversal potential of GABA and a decrease in the conductance of presynaptic GABA, which are both regulated by brain-derived neurotrophic factor.

## Graphical abstract

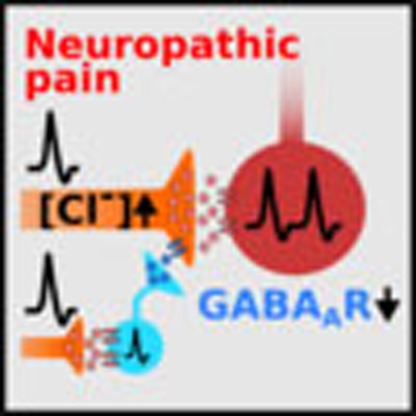

It has been almost half a century since the gate control theory of pain was first proposed by Melzack and Wall^1^. This theory suggests that nociceptive sensory information transmitted to the brain is normally under strong pre- and postsynaptic inhibitory control in the spinal cord. The gate control theory further predicts that under pathological conditions a disruption of the spinal inhibition efficacy may contribute to chronic pain development. Both pre- and postsynaptic inhibitions are controlled by local inhibitory interneurons and inhibitory descending fibres^2^. GABA_A_ receptors (GABA_A_R) located in primary afferent terminals of nociceptors cause reduction in transmitter release, thus modulating the afferent input from dorsal root ganglion (DRG) neurons into nociceptive-specific projection neurons (presynaptic inhibition), whereas postsynaptic GABA_A_R in the spinal cord neurons directly reduce their excitability and therefore control the output (postsynaptic inhibition). Although there is growing evidence indicating that spinal inhibition contribute to physiological and pathological pain sensation^2^,^3^,^4^,^5^,^6^,^7^,^8^, the precise contribution of different mode of inhibition (pre versus post) in various pain modalities remains elusive. This is mainly due to the difficulty to selectively manipulate pre- or postsynaptic inhibition^9^.

Unlike neurons in adult central nervous system, under physiological conditions, DRG neurons have a rather high *E*_GABA_, which determines a depolarizing effect by GABA_A_R activation. This primary afferent depolarization (PAD) is involved in the presynaptic inhibitory control of pain possibly through inactivation of voltage-gated sodium or calcium channels and through activation of a shunting conductance, which ultimately inhibits the propagation of action potential into presynapse, thus the transmitter release^6^,^10^. It has been proposed that under pathological conditions, such as tissue or nerve injury, a depolarizing shift of *E*_GABA_ in DRG neurons, which may result in an enhanced PAD sufficient to directly evoke an action potential, would lead to an excitatory effect of presynaptic GABA_A_R activation and cause touch-evoked pain^11^,^12^. Indeed, after nerve injury, a depolarizing shift of *E*_GABA_ in DRG neurons has been observed^13^,^14^. However, it remains unclear whether this shift switches presynaptic inhibition into excitation and contributes to neuropathic pain.

Recently, the loss of postsynaptic inhibition caused by brain-derived neurotrophic factor (BDNF) has been shown as an important substrate for neuropathic pain^15^,^16^. Peripheral tissue or nerve damage increases the expression of BDNF in DRG and the spinal cord^17^,^18^. A growing body of evidence has demonstrated that BDNF binding to its high-affinity receptor, TrkB receptor, is involved in both induction and maintenance of neuropathic pain^19^,^20^. BDNF released from activated microglia in the spinal cord dorsal horn binding to TrkB receptors on nociceptive-specific projection neurons causes downregulation of the neuronal potassium-chloride cotransporter KCC2, impairing Cl^−^ homeostasis and leading to a depolarizing shift in *E*_GABA_. This shift diminishes the postsynaptic inhibition mediated by GABA_A_R. Both presynaptic primary afferent fibres and postsynaptic neurons in dorsal horn of the spinal cord have been shown to express full-length TrkB receptor^21^,^22^. However, although the diminishing effect of postsynaptic BDNF-TrkB signalling after injury on postsynaptic inhibition has been well characterized, the consequence of BDNF binding to presynaptic TrkB receptors and the effect on presynaptic inhibition remains elusive.

Here we show that immediately after nerve injury there is a transient depolarizing shift in *E*_GABA_ and a reduction in the conductance of presynaptic GABA_A_R (*G*_GABA_) in DRG neurons, together leading to a reduction of presynaptic inhibition but not a switch to excitation. Two-photon imaging of calcium signal explicitly in primary afferent terminals in the spinal cord confirms the reduction of GABA-mediated presynaptic inhibition. Exogenous BDNF mimics the alteration in both GABA_A_R function and the neuropathic pain syndrome, whereas blocking BDNF and its receptor TrkB signalling reverse the change of GABA_A_R function and the reduced nociceptive threshold after nerve injury. To specifically determine the function of presynaptic inhibition in pain behaviour, we engineered mice deficient in GABA_A_R in primary nociceptors. These mice display increased sensitivity to both mechanical and thermal stimuli. Unlike the wild-type (WT) littermates, the thermal hypersensitivity in these mice cannot be further sensitized by nerve lesions or BDNF. Our results provide the first *in vivo* evidence that presynaptic inhibition is required for setting the sensitivity of pain behaviour under physiological condition and indicate that after nerve injury BDNF-modulated loss of presynaptic inhibition, but not switch to excitation, is crucial for the neuropathic pain initiation.

## Results

### Transient disinhibition in DRG neurons after nerve injury

**Figure 1:**
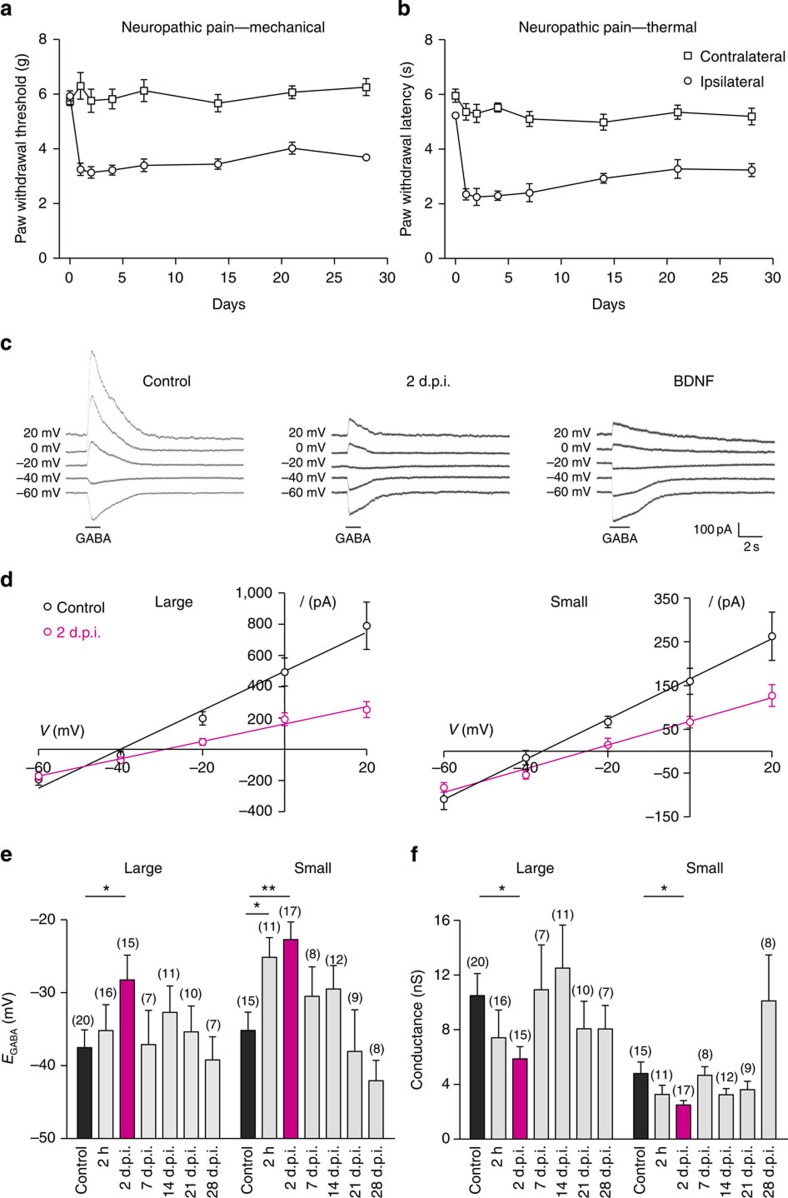
CCI induced a transient depolarizing shift of *E*_GABA_ and reduction of *G*_GABA_ in primary sensory neurons. Mechanical (**a**) and thermal hyperalgesia (**b**) induced by CCI surgery on the left sciatic nerve. *n*=7 mice (two-way analysis of variance; *P*<0.0001 for both). (**c**) Representative traces of GABA-activated currents recorded in DRG neurons during gramicidin perforated patch at various holding potential mV. (**d**) Current–voltage relationship for GABA-activated response from large (left) and small (right) neurons. (**e**) Bar graph showing the time course of the changes in *E*_GABA_. (**f**) Bar graph showing the time course of the changes in conductance of GABA_A_R. The number of neurons recorded is indicated in parentheses in each panel. *P<0.05; **P<0.01; unpaired *t*-test. Error bars indicate s.e.m.

To investigate the alteration of GABA_A_R after nerve injury, we first induced neuropathic pain in mice by making chronic constriction injury (CCI) to the sciatic nerve (Fig. 1a,b). We observed that both mechanical and thermal hypersensitivity had reached the peak effect 2 days post injury (d.p.i.) and persisted for at least 4 weeks tested. Therefore, we decided to first measure the *E*_GABA_ of DRG neurons prepared from 2 d.p.i. or intact animals by performing gramicidin-perforated patch recordings. We analysed the data separately for large (>25 μm diameter, putative mechanoreceptors) and small (≤25 μm diameter, putative nociceptors) neurons (Fig. 1c–f)^23^. Responses to exogenous GABA (γ-aminobutyric acid) application showed that *E*_GABA_ was −37.5±2.4 mV in control large neurons (diameter: 29.0±0.6 μm; *n*=20) and −35.2±2.5 mV in small neurons (diameter: 21.4±0.8 μm, *n*=15). Two days post nerve injury, a significant depolarization shift of *E*_GABA_ was observed in both groups of neurons (Fig. 1d,e). In large neurons (29.8±0.8 μm, *n*=15) *E*_GABA_ shifted to −28.3±3.4 mV (*P*<0.05) and in small neurons, which often respond to noxious stimuli (20.4±0.7 μm, *n*=17), *E*_GABA_ was −22.7±2.4 mV (*P*<0.01). To determine whether this shift persists as long as the neuropathic pain syndrome, we further measured the *E*_GABA_ at different time points after CCI. Surprisingly, we found this depolarizing shift occurred immediately (2 h) after nerve injury but already started to recover 7 d.p.i. and reached control levels at 21 d.p.i., although the neuropathic pain symptoms still persisted (Fig. 1e). Thus, after nerve injury, the *E*_GABA_ in both small and large neurons underwent a transient depolarizing shift, which might result in an enhanced PAD sufficient to directly evoke an action potential, indicating a transient reduction of GABA_A_R-mediated presynaptic inhibition or even a switch to excitation.

**Figure 2:**
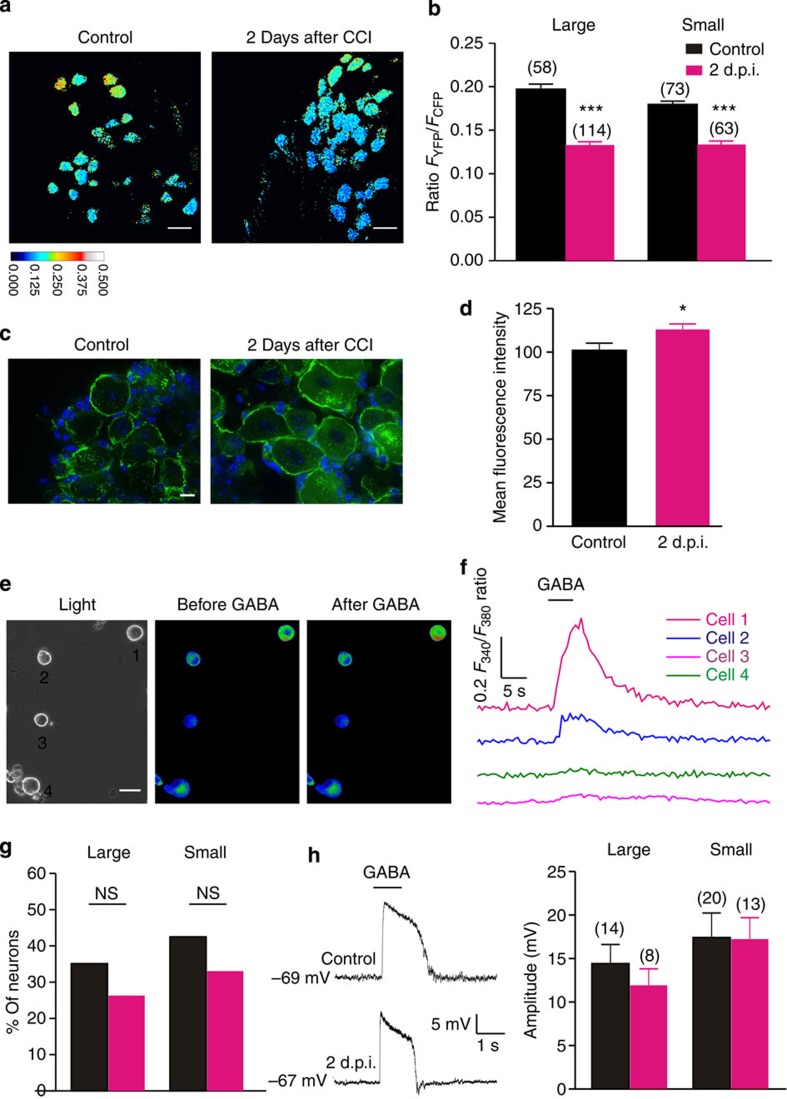
*E*_GABA_ shift after CCI is due to the elevated intracellular chloride regulated by NKCC1. (**a**) Two-photon images of Clomeleon in acutely dissected DRGs from intact (left) and 2 days post injury (right) animals. (**b**) Decrease in Clomeleon ratio (*F*_YFP_/*F*_CFP_) in intact DRG compared with 2 days after injury, corresponding to an increase in intracellular chloride (unpaired *t*-test, *P*<0.0001). (**c**) Representative staining of NKCC1 (green) on the cell membrane of DRG from control and 2 d.p.i. animals. Nuclei (blue) were stained with DAPI (4,6-diamidino-2-phenylindole). (**d**) Quantification of immunofluorescence signals for cell surface NKCC1 protein (*n*=3 DRGs from 3 animals for each; unpaired *t*-test, *P*<0.05). (**e**) Bright-field and Fura-2 ratiometric images of DRG neurons labelled as 1–4. (**f**) Representative traces evoked by GABA (1 mM) in the calcium imaging experiment from the four neurons labelled in **e**. Δ*F*_340_=*F*_340_–*B*_340_, Δ*F*_380_=*F*_380_–*B*_380_. (**g**) The proportion of neurons displaying a GABA-evoked in [Ca^2+^]_*i*_ was not different between control and injured animals (control: large neuron: *n*=37, small *n*=207; 2 d.p.i.: large neuron: *n*=19, small *n*=127; Fisher’s test, *P*>0.05). (**h**) Left, representative traces recorded by perforated patch under current clamp. Right, the amplitude of depolarization induced by brief GABA application was not different between control and injured animals (unpaired *t*-test, *P*>0.05). Error bars indicate s.e.m. Scale bars, (**a**) 50 μm; (**c**) 10 μm; (**e**) 30 μm. *P<0.05; ***P<0.001.

To confirm the *E*_GABA_ shift after nerve injury is due to the change of Cl^−^ homeostasis in DRG neurons, we performed two-photon Cl^−^ imaging in acutely isolated DRG from transgenic mouse carrying a chloride sensor Clomeleon. Two days after nerve injury, a decrease in Clomeleon ratio (yellow fluorescent protein (YFP)/cyan flurescent protein (CFP)), corresponding to an increase in intracellular chloride, was observed in both large and small neurons (*P*<0.0001 for both, Fig. 2a,b).

Previous reports have indicated that the Cl^−^ homeostasis in DRG neurons is mainly due to the sodium potassium chloride co-transporter (NKCC1)^13^,^24^,^25^; we therefore examined the effect of injury on DRG NKCC1 expression. We first analysed the change of NKCC1 protein level in whole DRG. Immunoblotting result showed a trend but not significant increase of NKCC1 expression (Supplementary Figs 1 and 2). NKCC1 activity is largely determined by the amount of NKCC1 proteins present on the plasma membrane and post-translational modifications. Therefore, we next quantified the NKCC1 membrane proteins in DRG neurons by performing immunohistochemistry and found the cell surface NKCC1 level was significantly higher in DRG neurons 2 days after injury than in control (Fig. 2c,d). This result demonstrates that NKCC1 is responsible for the increase in [Cl^−^]_i_ induced by nerve injury.

In vertebrate primary afferent axons, activation of presynaptic GABA_A_R causes depolarization and increases membrane conductance (shunting). It has been shown that either PAD or shunting is sufficient to produce inhibition^26^. To examine the change of shunting effect, we calculated the peak *G*_GABA_ from the plot of GABA-induced peak current versus holding potential (Fig. 1d,f). The GABA-mediated conductance in large neurons (12.0±2.0 ns, *n*=20) was significantly higher than in small neurons (4.8±0.8 ns, *n*=15, *P*<0.005) taken from naive animals. Two days after injury, there was a significant reduction in both large (6.1±0.9 ns, *n*=15, *P*<0.05) and small neurons (2.5±0.3 ns, *n*=17, *P*<0.05) (Fig. 1f). This result is consistent with previous reports that GABA-mediated current strength decreased and messenger RNA of GABA_A_R was significantly downregulated in DRG neurons after injury^3^,^27^,^28^. Similar to the time course of *E*_GABA_ shift, significant reduction of GABA-mediated conductance was only detected 2 days after injury and started to recover from 1 week on (Fig. 1f), indicating a transient reduction of shunting-mediated presynaptic inhibition.

Taken together, the early transient shift of *E*_GABA_ and reduction of *G*_GABA_, both lead to the same alteration of GABA_A_R function after nerve injury, that is, a loss of inhibitory effect. It has been reported that after nerve injury, the shift of *E*_GABA_ could switch postsynaptic inhibition into excitation^16^. This raised the question here whether the depolarizing shift of *E*_GABA_ in presynaptic primary sensory neurons would also lead to excitatory effect, while the *G*_GABA_ is reduced. To address this, we first performed Ca^2+^ imaging of Fura-2-AM-loaded DRG neurons from intact and 2 d.p.i. animals. Exogenous GABA caused an intracellular Ca^2+^ concentration increase in 35% (*n*=37) of large neurons and 43% (*n*=207) of small neurons. Two days after nerve injury, unlike the postsynaptic GABA_A_R activation^16^, this proportion did not increase (Fig. 2e–g, *P*>0.05 for both large and small neurons). This result suggests that after nerve injury the GABA_A_R activation did not induce Ca^2+^ influx in more neurons and become excitatory. This is mostly because accompanying the depolarizing shift of *E*_GABA_ there is a reduction of *G*_GABA_, which might limit the increase of PAD. We further explored this idea more directly by performing gramicidin-perforated patch recordings under current clamp mode. Administration of exogenous GABA induced a similar depolarization in DRG neurons from both intact and injured animals (Fig. 2h). In addition, only in a few neurons (control: 3 of 14 large and 2 of 20 small neurons; 2 d.p.i.: 0 of 8 large and 1 of 14 small neurons) could an action potential be directly evoked by GABA application, indicating a non-excitatory effect of GABA_A_R activation. Thus, our results here showed that after nerve injury, unlike postsynaptic disinhibition, presynaptic inhibition is not switched to excitation but rather abolished via reduction of the shunting effect. The similar phenomena has been reported by Wei and colleagues (2013) in a trigeminal neuropathic pain model where they observed a disinhibition and excitation effect on pre- and postsynaptic GABA actions, respectively^29^.

### Loss of presynaptic inhibition after injury

**Figure 3:**
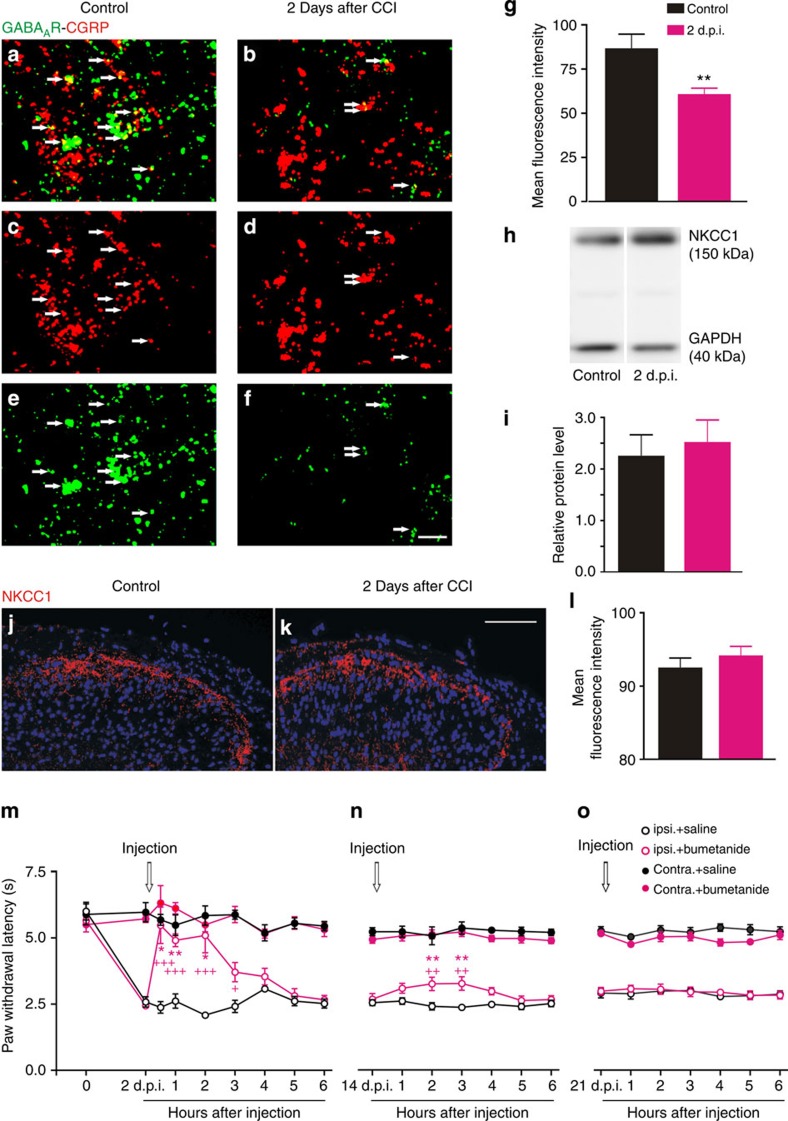
GABA_A_R and NKCC1 expression in the spinal dorsal horn. (**a**–**f**) Co-localization of α1-GABA_A_R (green) with calcitonin gene-related peptide (CGRP)-positive terminals (red) in parasagittal sections of lumbar spinal cord of control and 2 d.p.i. mice. Arrows mark representative double-labelled terminals. (**g**) Quantification of the α1-GABA_A_R fluorescence intensity in the CGRP-positive areas in the dorsal horn of control and 2 d.p.i. mice (control: *n*=8 sections (2 mice); 2 d.p.i.: *n*=31 sections (3 animals); unpaired *t*-test, *P*<0.01). (**h**) Western blotting showing the specific band of NKCC1 in control and 2 d.p.i. spinal cord. *GAPDH* was used as housekeeping gene. (**i**) Quantification of the bands intensities relative to GAPDH expression (*n*=6 ipsilateral spinal cord (L4–L5) from injured animals and 6 halves spinal cord (L4–L5) from control animals. Three western blotting were performed in total; unpaired *t*-test, *P*>0.05). (**j**,**k**) Representative staining of NKCC1 (red) in control and 2 d.p.i. spinal cord dorsal horn. Nuclei (blue) were stained with DAPI. (**l**) Quantification of immunofluorescence signals for NKCC1 (*n*=3 sections of each spinal cord from three control and three 2 d.p.i. animals; unpaired *t*-test, *P*>0.05). Intraperitoneal administration of NKCC1 inhibitor bumetanide or saline 2 days (**m**), 14 days (**n**) or 21 days (**o**) after nerve injury, *n*=6 mice per group (bumetanide effect: one-way analysis of variance (ANOVA) with *post-hoc* Dunnett’s test; 2 d.p.i., *P*=0.001; 14 d.p.i., *P*=0.0001; 21 d.p.i., *P*>0.05. Ipsilateral+saline versus ipsilateral+bumetanide, two-way ANOVA with *post-hoc* Bonferroni’s test, 2 d.p.i., *P*=0.0001; 14 d.p.i., *P*<0.05; 21 d.p.i., *P*>0.05.). Scale bars: (**a**–**f**), 2 μm; (**j**,**k**), 100 μm (scale bar only shown in **f** and **k**). *P<0.05; **P<0.01, versus control (**g**), or versus before injection (**m**,**n**); ^+^*P*<0.05; ^++^*P*<0.01; ^+++^*P*<0.001 versus ipsilateral+saline (**m**,**n**). Error bars indicate s.e.m.

Although cultured DRG neurons have long been used as a model in evaluating the pathogenic mechanisms of peripheral neuropathies^30^, does the shift of *E*_GABA_ and reduction of *G*_GABA_ on the soma of DRG neurons we observed here really represent what happens in the primary afferent central terminal, where presynaptic inhibition locates, after nerve injury? To answer this question, we double stained spinal cord sections with antibodies to α1-GABA_A_R and calcitonin gene-related peptide and found that indeed 2 days after nerve injury there is a significant reduction of presynaptic GABA_A_R expression (Fig. 3a–g). Furthermore, we have checked the NKCC1 expression level on the primary afferent central terminal after nerve injury. Both immunoblotting and immunohistochemical staining results showed a trend but not significant increase of NKCC1 (Fig. 3h–l and Supplementary Fig. 3). It has been shown that a nerve injury induced increase of the phosphorylated NKCC1 in DRG accounts for the intracellular Cl^−^ accumulation^13^,^31^. This might explain why we could not detect an increase of overall NKCC1 expression level in either whole DRG or the spinal cord. To further confirm the involvement of NKCC1 in the hypersensitive behaviour developed after nerve injury, we have treated 2, 14 and 21 d.p.i. animals with either saline or NKCC1 inhibitor bumetanide. Administration of bumetanide significantly increased the paw withdrawal latency time in 2 d.p.i. mice. This anti-hyperalgesic effect of bumetanide became much less profound in 14 d.p.i. mice and was completely absent in 21 d.p.i. mice, confirming that a transient upregulation of NKCC1 activity after nerve injury contributes to neuropathic pain initiation (Fig. 3m–o).

**Figure 4:**
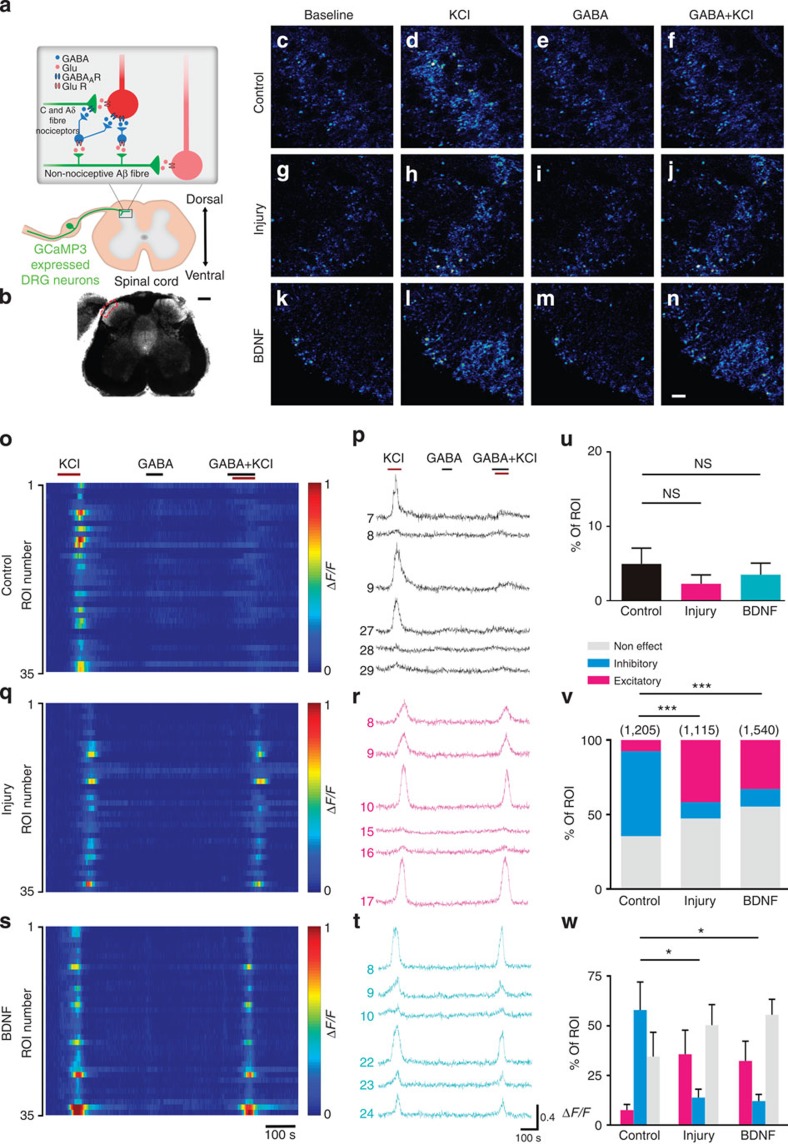
Two-photon calcium imaging in primary afferent terminals. (**a**) Scheme illustrating the specific expression pattern of GCaMP3 in primary sensory neurons. (**b**) Bright-field picture of spinal cord slice used for two-photon imaging. (**c**–**n**) Two-photon images from control, injured and BDNF-treated mouse spinal cord slice were showed in first (**c**–**f**), second (**g**–**j**) and third row (**k**–**n**), respectively. (**o**,**q**,**s**) Relative GCaMP3 fluorescence changes (Δ*F*/*F*) during high KCl, GABA alone and GABA+KCl in afferent terminals are shown as colour maps. (**p**,**r**,**t**) Traces showing the relative change of GCaMP3 fluorescence during high KCl, GABA and GABA+KCl in example ROIs from control (**p**), injured (**r**) and BDNF (**t**)-treated mouse spinal cord slice. (**u**) Fraction of ROIs responded to GABA application from separate experiments (control: *n*=4; injury, *n*=5; BDNF, *n*=6). Error bars indicate s.e.m. (**v**) and (**w**) Frequency histograms of the proportion of three different effects of GABA on high KCl-induced GCaMP3 fluorescence increase. (**v**) The proportion from all ROIs pooled from all experiments. The number of ROIs recorded is indicated in parentheses in each panel. (Control versus injury, *χ*^2^-test, *P*<0.0001; control versus BDNF, *χ*^2^-test, *P*<0.0001). Error bars indicate s.e.m. Error bars in **w** represents the s.e.m. of the proportion of ROIs from separate experiments (experiment number: control, *n*=5; injury, *n*=6; BDNF, *n*=7; inhibitory effect: control versus injury, unpaired *t*-test, *P*<0.05; control versus BDNF, unpaired *t*-test, *P*<0.05. excitatory effect: control versus injury, unpaired *t*-test, *P*>0.05; control versus BDNF, unpaired *t*-test, *P*>0.05). Scale bars, (**b**) 200 μm; (**c**–**n**) 10 μm; (scale bar only shown in **b** and **n**). *P<0.05; ***P<0.001.

The final consequence of presynaptic inhibition is to reduce the calcium influx, thus the transmitter release from presynapses. To investigate the functional change of presynaptic GABA_A_R in the spinal cord after nerve injury, we sought to perform two-photon calcium imaging specifically in the primary afferent terminals. To target genetically encoded calcium sensors to primary afferents, we crossed mice carrying the floxed *GCaMP3* allele (Ai38)^32^ to a mouse line expressing Cre recombinase from the locus of the sensory neuron-specific gene *Advillin* (advillin-Cre)^33^ (Fig. 4a,b and Supplementary Fig. 4a,b). To record the calcium transients in the primary afferent terminals, we performed two-photon imaging on the spinal cord slices taken from control or 2 d.p.i. mouse. Direct application depolarizing concentration of KCl to the spinal cord slice elicited robust fluorescence increases in the primary afferent terminals from both control and 2 d.p.i. mice (Fig. 4c,d,g,h,o–r and Supplementary Movies 1 and 2). Next, we applied GABA to test whether GABA alone could be excitatory and induce calcium transient. In either the control or the 2 d.p.i. spinal cord slices, we did not observe much excitatory effect of GABA (control: 5.0±2.1%, 712 regions of interest (ROIs) from 4 slices from 4 animals; injury: 2.3±1.2%, 566 ROIs from 5 slices from 5 animals; *P*>0.05; Fig. 4e,i,o–r,u). Consistent with our finding on the cultured DRG neurons, this result suggests that after nerve injury the activation of presynaptic GABA_A_R alone does not become excitatory, not sufficient enough to directly induce Ca^2+^ influx, thus the transmitter release from presynapses. Finally, to test the inhibitory effect of GABA on presynaptic activity, we applied GABA together with depolarizing concentration of KCl. GABA exhibited an inhibitory effect on KCl-induced fluorescence increase in only 11% of the ROIs from injured animals, in contrast with 57% of the ROIs from control mice (control: 1,205 ROIs from 5 slices from 5 animals; injury: 1,115 ROIs from 6 slices from 6 animals; *P*<0.0001; Fig. 4f,j,o–r,v–w), indicating a substantial loss of presynaptic inhibition after nerve injury.

### Abnormal pain behaviour in *sns- β3* ^−*/*−^mouse

**Figure 5:**
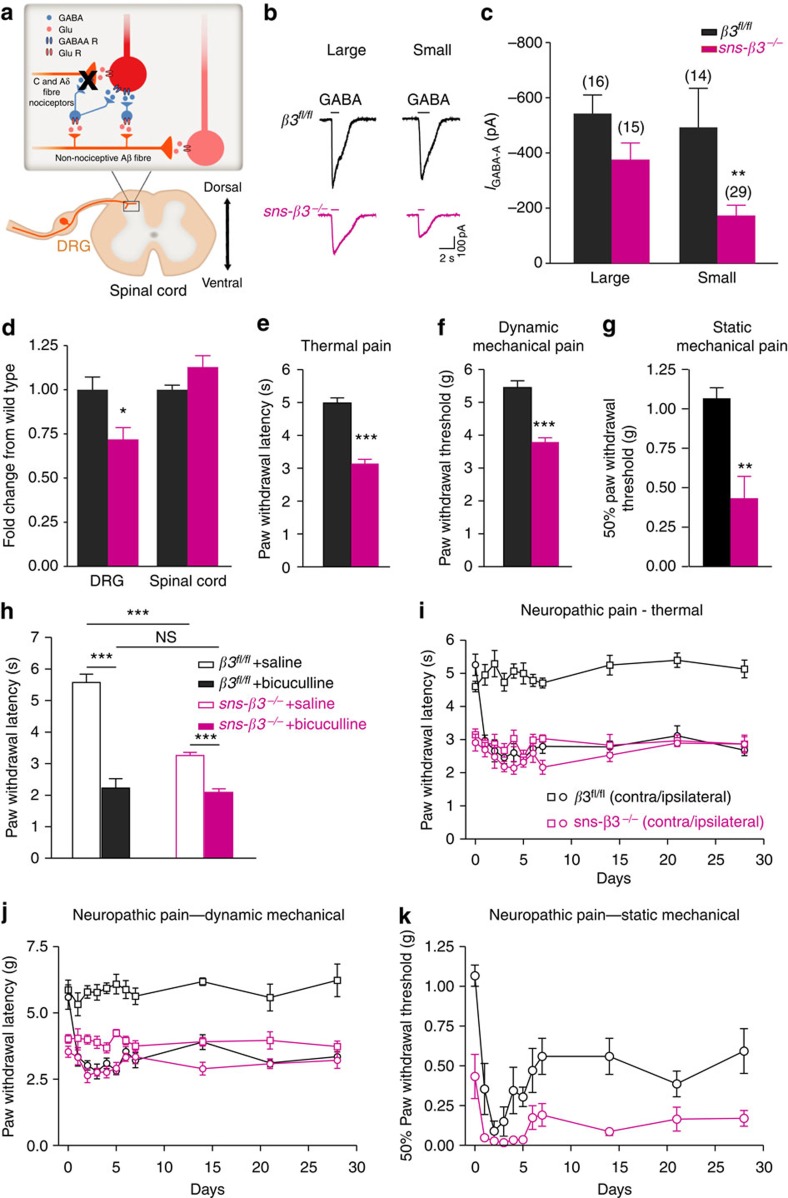
Pain behaviour in nociceptor-specific presynaptic GABA_A_R knockout (KO) mice and littermate controls. (**a**) Scheme illustrating the specific KO of GABA_A_R in nociceptors. (**b**) Representative traces of GABA-activated currents recorded in large (left) and small (right) DRG neurons clamped at −60 mV from *β3*^*fl/fl*^ and *sns- β3*^−*/*−^ mice. (**c**) Statistical analysis shows the amplitude of GABA-activated current is significantly reduced in small but not in large DRG neurons from *sns- β3*^−*/*−^ mice compared with *β3*^*fl/fl*^ mice; large neuron: *P*>0.05; small neuron: *P*<0.01, unpaired *t*-test. (**d**) Quantification of *gabaβ3* gene transcripts in the DRGs and spinal cord of *sns- β3*^−*/*−^ and their WT littermates. DRG: *P*<0.05; spinal cord: *P*>0.05; *n*=9 for each. (**e**) Hargreaves: *sns- β3*^−*/*−^ mice exhibit higher sensitivity to noxious heat stimulation (*β3*^*fl/fl*^: *n*=12; *sns- β3*^−*/*−^: *n*=12. *P*<0.0001). (**f**) Dynamic Plantar Aesthesiometer: *sns- β3*^−*/*−^ mice show increased sensitivity to noxious mechanical stimulation (*β3*^*fl/fl*^: *n*=12; *sns- β3*^−*/*−^: *n*=12. *P*<0.0001). (**g**) Von Frey: mechanical threshold were measured using Von Frey hairs (*β3*^*fl/fl*^: *n*=6; *sns- β3*^−*/*−^: *n*=6. *P*<0.01). (**h**) Bicuculline increased thermal sensitivity of both *sns- β3*^−*/*−^ and their WT littermates. *n*=6 mice per group (*β3*^*fl/fl*^: saline versus bicuculline, *P*<0.0001; *sns- β3*^−*/*−^: saline versus bicuculline, *P*<0.0001; saline: *β3*^*fl/fl*^ versus *sns- β3*^−*/*−^, *P*<0.0001; bicuculline: *β3*^*fl/fl*^ versus *sns- β3*^−*/*−^, *P*>0.05). (**i**) *sns- β3*^−*/*−^ mice failed to develop thermal hypersensitivity after CCI. *n*=6 mice per group. (Two-way analysis of variance (ANOVA): *β3*^*fl/fl*^ contralateral versus ipsilateral, *P*<0.0001; *sns- β3*^−*/*−^ contralateral versus ipsilateral, *P*=0.0457; *β3*^*fl/fl*^ ipsilateral versus *sns- β3*^−*/*−^ ipsilateral after injury, *P*>0.05). (**j**) Dynamic mechanical hypersensitivity is significantly reduced in *sns- β3*^−*/*−^ mice. *n*=6 mice per group. (Two-way ANOVA: *β3*^*fl/fl*^ contralateral versus ipsilateral, *P*<0.0001; *sns- β3*^−*/*−^ contralateral versus ipsilateral, *P*=0.0004; *β3*^*fl/fl*^ ipsilateral versus *sns- β3*^−*/*−^ ipsilateral after injury, *P*>0.05). (**k**) Static mechanical allodynia is not affected in *sns- β3*^−*/*−^ mice. *n*=6 mice per group (Two-way ANOVA, *P*<0.0001). Error bars indicate s.e.m. *P<0.05; **P<0.01; ***P<0.0001.

Although we have shown that after nerve injury there is an early transient shift of *E*_GABA_ and reduction of *G*_GABA_ leading to a reduced presynaptic inhibition, its relevance to the induction of neuropathic pain behaviour remains unclear. To address the functional importance of GABA_A_R-mediated presynaptic inhibition *in vivo* and determine whether presynaptic disinhibition is involved in neuropathic pain induction, we generated conditional nociceptor-specific β3-GABA_A_R knockout mice (*sns- β3*^−*/*−^) by crossing mice carrying the floxed *Gabrb3* allele (Gabrb3^*fl/fl*^) to a mouse line expressing Cre recombinase under the control of the nociceptor-specific sodium channel Na_v_1.8 promotor (SNS-Cre)^9^,^34^ (Fig. 5a).

To quantify changes in GABA_A_R β3 subunit expression, we performed quantitative reverse transcriptase–PCR in lumbar DRG and the spinal cord. Significant reduction of β3 subunit expression was observed in DRG but not in the spinal cord when *sns- β3*^−*/*−^ mice were compared with their WT littermates (Fig. 5d). To confirm that conditional genetic deletion of β3 subunit attenuated GABA_A_R function in DRG neurons, whole cell voltage-clamp recordings were performed on DRG neurons isolated from *sns- β3*^−*/*−^ mice or their WT littermates. Exogenous GABA-mediated currents were significantly reduced in small DRG neurons from *sns- β3*^−*/*−^ mice compared with WT mice (large neurons: −375±60 pA, *n*=15 versus −542±67 pA, *n*=16; *P*>0.05; small neurons: −173±37 pA, *n*=29 versus −492±141 pA, *n*=14; *P*<0.01), demonstrating a functional loss of GABA_A_R in DRG neurons isolated from *sns- β3*^−*/*−^ mice (Fig. 5b,c).

Next we analysed pain behaviour in *sns- β3*^−*/*−^ mice. We first examined acute heat and mechanical nocifensive reflex. *sns- β3*^−*/*−^ adult male mice exhibited a hypersensitive phenotype to both noxious heat (Hargreaves test) and mechanical stimulation (Dynamic plantar and von Frey test) compared with their WT male littermates (Fig. 5e–g), indicating that the presynaptic GABA_A_R is required to control both heat and mechanical pain signal processing under healthy condition. In contrast, acute sensitivity to cold stimulus is not affected in *sns- β3*^−*/*−^ mice as demonstrated by cold plantar test (Supplementary Fig. 5a). This is, to our knowledge, the first *in vivo* evidence showing that presynaptic inhibition plays a major role in the processing of noxious signals^2^. By contrast, no significant difference was observed in the hot plate test between WT and *sns- β3*^−*/*−^ mice (Supplementary Fig. 5b), which is consistent with observations in global *β3*^−*/*−^ mice^35^. Hot plate test is considered to be supraspinally integrated responses. The lack of difference might be due to the adjustment of pain perception at the cognitive level during development.

Both pre- and postsynaptic disinhibition have been proposed to be involved in neuropathic pain initiation and maintenance^2^. If the hypersensitivity developed after injury is due to mechanisms including postsynaptic disinhibition other than loss of presynaptic inhibition, we should expect the same extent of increase in heat or mechanical sensitivity in the *sns- β3*^−*/*−^ mice as in WT mice. Strikingly, when tested with CCI pain model, the heat sensitivity in *sns- β3*^−*/*−^ mice did not increase, while the injury produced profound thermal hypersensitivity in WT littermates (Fig. 5i). After nerve injury, the paw withdrawal latency in WT littermates decreased to the level of *sns- β3*^−*/*−^ mice. To test the possibility that the loss of thermal hypersentivity development in *sns- β3*^−*/*−^ mice after injury is due to the floor effect, we administered bicuculline or saline to the *sns- β3*^−*/*−^ mice and their WT littermates. Bicuculline administration dramatically increased the heat sensitivity in both WT and *sns- β3*^−*/*−^ mice (Fig. 5h), excluding the possibility of floor effect in *sns- β3*^−*/*−^ mice. This indicates that loss of presynaptic inhibition is sufficient to induce thermal hypersensitivity after nerve injury. Dynamic mechanical hypersensitivity was also significantly reduced in *sns- β3*^−*/*−^ mice when compared with their WT littermates (Fig. 5j).

The switch of presynaptic inhibition to excitation has been proposed as one of the mechanisms underlying touch-evoked pain^12^. To test this type of mechanical hypersentivity, we performed a von Frey filament test. After nerve injury, both *sns- β3*^−*/*−^ mice and their WT littermates showed a significant reduction in the mechanical threshold for evoking a nociceptive withdrawal reflex (Fig. 5k). Despite the fact that *sns- β3*^−*/*−^ mice are more sensitive to mechanical stimuli under healthy conditions (Fig. 5g), they developed the same extent of static mechanical allodynia as their WT littermates after nerve injury. Together with our two-photon calcium imaging result on the primary afferents, these findings demonstrate that nociceptor-specific presynaptic GABA_A_R-mediated inhibition does not become excitatory and contribute to static mechanical allodynia after nerve injury. Factoring this together with the transient change of GABA_A_R function after nerve injury, we conclude that loss of presynaptic inhibition is required for the generation but not for the maintenance of thermal and mechanical hyperalgesia induced by nerve injury, while postsynaptic disinhibition contributes mostly to the development of static mechanical allodynia.

### Presynaptic disinhibition regulated by BDNF

**Figure 6:**
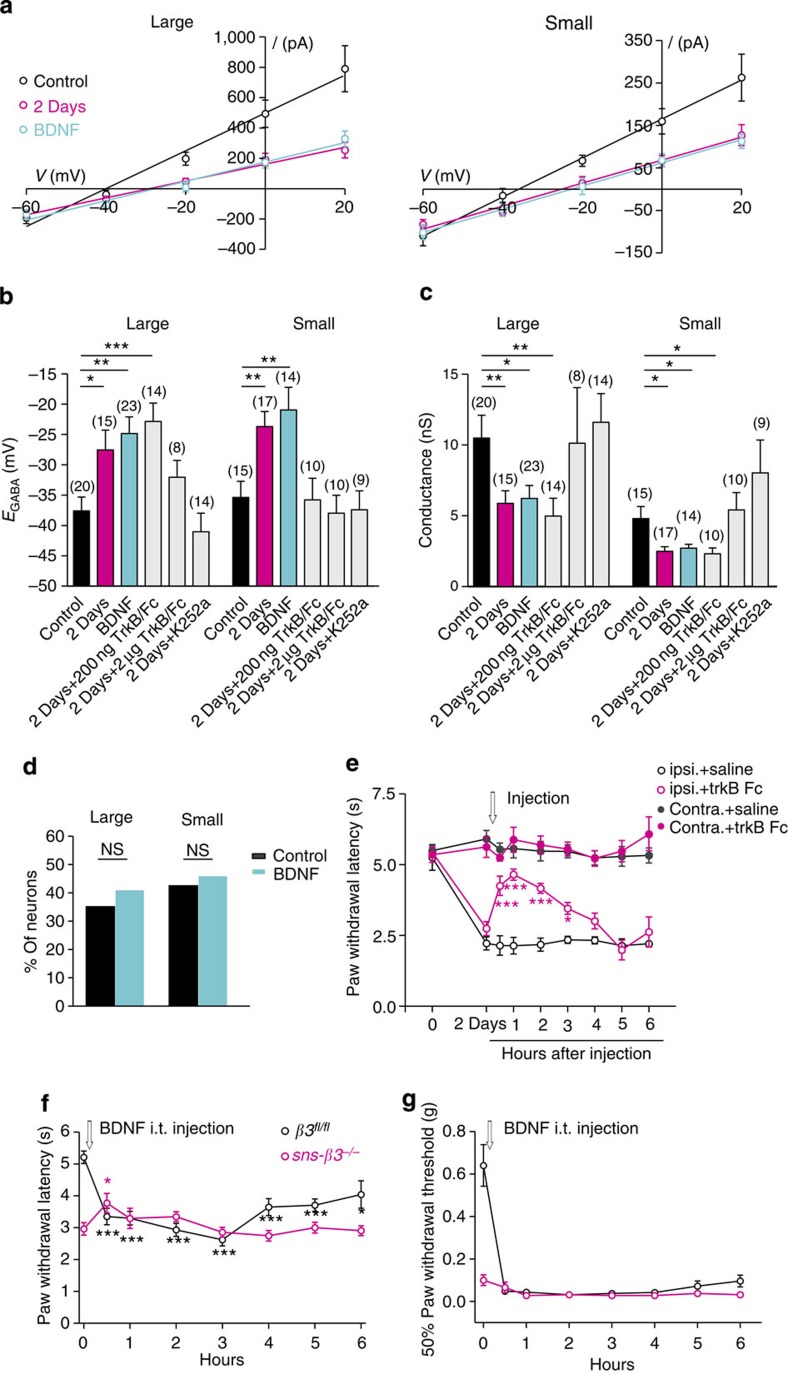
BDNF contributes to neuropathic pain induction through presynaptic GABA_A_R. (**a**) Current–voltage relationship for GABA-activated response from large (left) and small (right) neurons. (**b**) BDNF mimics the effect of nerve injury on *E*_GABA_ and functional inhibition of BDNF-TrkB signalling reverses the depolarizing shift in *E*_GABA_ (the number of neurons recorded is indicated in parentheses in each panel; unpaired *t*-test). (**c**) BDNF mimics the effect of nerve injury on *G*_GABA_ and functional inhibition of BDNF-TrkB signalling reverses the reduction in *G*_GABA_ (unpaired *t*-test). (**d**) The proportion of neurons displaying a GABA-evoked increase in [Ca^2+^]_*i*_ was not different between control and BDNF-treated DRG neurons (control: large neuron: *n*=37, small *n*=207; BDNF: large neuron: *n*=22, small *n*=85; Fisher’s test, *P*>0.05). (**e**) Intrathecal administration of TrkB-Fc 2 days after nerve injury caused a significant increase in the paw withdrawal latency. *n*=6 mice/group (TrkB-Fc effect: two-way analysis of variance (ANOVA) with *post-hoc* Bonferroni test; ipsilateral+saline versus ipsilateral+TrkB-Fc, *P*<0.0001). Intrathecal injection of BDNF failed to induce heat hyperalgesia (**f**) in *sns- β3*^−*/*−^ mice (*n*=6 mice per group, BDNF effect: one-way ANOVA with *post-hoc* Dunnett’s test; *β3*^*fl/fl*^, *P*<0.01; *sns- β3*^−*/*−^, *P*>0.05; *β3*^*fl/fl*^ versus *sns- β3*^−*/*−^, two-way ANOVA, *P*<0.001), but did not affect the development of static mechanical allodynia (**g**) (*n*=6 mice/group, one-way ANOVA with *post-hoc* Bonferroni test; *β3*^*fl/fl*^, *P*<0.0001; *sns- β3*^−*/*−^, *P*<0.0001; *β3*^*fl/fl*^ versus *sns- β3*^−*/*−^, two-way ANOVA, *P*<0.0001). *P<0.05; **P<0.01; ***P<0.001 versus control (**b**,**c**), or versus before injection (**e**,**f**). Error bars indicate s.e.m.

We next asked how presynaptic GABA_A_R function is controlled after nerve injury. BDNF is upregulated in both activated microglia and DRG neurons after nerve injury^15^,^18^,^36^. If the depolarizing shift of *E*_GABA_ and decrease in *G*_GABA_ is due to BDNF^37^, an exogenous BDNF treatment on DRG neurons from a naive mouse should have the same effect. To test this possibility, we incubated control DRG neurons with BDNF. *E*_GABA_ of cultured DRG neurons incubated with BDNF (50 ng ml^−1^) overnight was significantly more depolarized than that of control neurons (large neuron: −24.9±2.7 mV, 30.8±0.8 μm, *P*<0.005, *n*=23; small neurons: −20.0±3.7 mV, 20.1±0.7 μm, *P*<0.005, *n*=14) (Figs 1c and 6a,b). In addition, a significant reduction of the conductance in large and small neurons was also observed (large neuron: 6.2±0.9 nS, *P*<0.05; *n*=23; small neurons: 2.7±0.3 nS, *P*<0.05, *n*=14) (Fig. 6a,c). Similar to what we found in cultured DRG neurons after injury, BDNF did not increase the GABA-mediated calcium influx (Fig. 6d). Thus, exogenous BDNF produced a similar effect as nerve injury on both *E*_GABA_ and *G*_GABA_ in both large and small neurons. We then carried out two-photon imaging experiments on BDNF-treated spinal cord slices (Fig. 4k–n,s–w and Supplementary Movie 3). A significant loss of inhibitory effect of GABA on KCl-induced fluorescence increase was observed, while GABA alone did not show any excitatory effect (Fig. 4s–w), confirming that BDNF is sufficient to cause loss of presynaptic inhibition but not switch to excitation after nerve injury.

If the altered GABA_A_R function after nerve injury is due to increased BDNF level in DRG and the spinal cord, a pharmacological blockade of BDNF signalling should be able to reverse the change of GABA_A_R function and the reduced nociceptive threshold after nerve injury. To examine this, we administered a BDNF-sequestering fusion protein (TrkB-Fc) intrathecally to the mice that had developed thermal hypersensitivity 2 days after CCI. A single injection of TrkB-Fc could significantly reverse the decrease in paw withdrawal latency, while saline produced no change (Fig. 6e). To determine whether BDNF-TrkB signalling is necessary for the nerve injury-induced change of presynaptic GABA_A_R function, we tested the effects of a TrkB-Fc and a Trk receptor inhibitor K252a on *E*_GABA_ and *G*_GABA_ in cultured large and small DRG neurons taken from 2 d.p.i. animals. Indeed, treatment with TrkB-Fc or K252a could completely reverse the *E*_GABA_ and *G*_GABA_ changes in both large and small neurons taken from injured animals (Fig. 6b,c). These findings indicate that BDNF-trkB signalling on DRG neurons is essential for the alteration in GABA_A_R function after nerve injury, which leads to neuropathic pain induction.

It has also been shown that blocking BDNF-TrkB signalling after injury by intrathecally administering anti-TrkB or TrkB-Fc could acutely reverse the shift of post-synaptic *E*_GABA_ in spinal neurons and the neuropathic pain symptoms^15^,^20^,^38^. However, both pre- and postsynaptic GABA_A_Rs are localized in the spinal cord and could be affected by intrathecal drug application. This raises the question as to whether the reduction in hyperalgesia behaviour is due to the reversal of pre- and/or postsynaptic disinhibition.

To discriminate the *in vivo* effect of BDNF on presynaptic inhibition from postsyatptic inhibition, we administered BDNF intrathecally to the *sns- β3*^−*/*−^ adult male mice or their WT male littermates (*n*=6 mice per group). A large body of evidence has demonstrated the similarities between long-lasting thermal or mechanical hypersensitivity induced by nerve injury and single intrathecal BDNF injection^38^,^39^,^40^. Indeed, we observed that BDNF could cause an increase in both thermal and mechanical sensitivity almost immediately after injection (0.5 h) and persist for at least 6 h of testing in WT mice (Fig. 6f,g). Remarkably, thermal hypersensitivity could not be further enhanced by BDNF in *sns- β3*^−*/*−^ mice (Fig. 6f), indicating that BDNF acts to remove nociceptor-specific presynaptic GABA_A_R-mediated inhibition to induce heat hypersensitivity. In contrast to the heat hypersensitivity, static mechanical allodynia development was reserved in *sns- β3*^−*/*−^ mice after BDNF injection (Fig. 6g), suggesting a presynaptic inhibition independent mechanism, most probably through postsynaptic disinhibition regulated by BDNF.

## Discussion

Here we have demonstrated that presynaptic inhibition is required for setting the pain sensitivity under physiological condition and its regulation by BDNF after nerve injury is essential for neuropathic pain initiation. Two distinct inhibitory mechanisms are employed in the spinal cord to control pain signal processing: presynaptic inhibition of the primary afferents and postsynaptic inhibition of the dorsal horn projection neurons. Enormous evidence has shown that blockade of spinal GABA-mediated inhibition produced an increase in nociceptive reactions to noxious stimuli and painful sensations to innocuous stimuli^41^,^42^,^43^,^44^,^45^,^46^. However, none of these studies made a clear distinction between pre- and postsynaptic inhibition. Zeilhofer’s laboratory has first applied a genetic approach to selectively knock out presynaptic GABA_A_R α2 subunit on spinal nociceptor terminals (*sns- α2*^−*/*−^ mice), to address the contribution of presynaptic inhibition to spinal pain control^9^. Surprisingly, GABAergic membrane currents recorded from nociceptive DRG neurons are the same between WT and *sns- α2*^−*/*−^ mice. Furthermore, *sns- α2*^−*/*−^ mice exhibited normal response thresholds to thermal and mechanical stimulation, and developed normal inflammatory and neuropathic pain. This is most probably due to the upregulation of other GABA_A_R subunits. Here we have generated a new mouse line in which GABA_A_R β3 subunit is selectively knocked out in primary nociceptors (*sns- β3*^−*/*−^ mice). The amplitude of GABA_A_R current is significantly reduced in *sns- β3*^−*/*−^ mice compared with WT mice, demonstrating a functional loss of presynaptic GABA_A_Rs. Remarkably, *sns- β3*^−*/*−^ mice exhibited a hypersensitive phenotype to both noxious heat and mechanical stimulation compared with WT mice. To our knowledge, this is the first *in vivo* evidence being able to dissect the relative contribution of presynaptic inhibition in controlling both heat and mechanical pain signal processing under healthy condition.

The capacity for neuronal inhibition by GABA_A_R activation is critically dependent on [Cl^–^]_i_. An increased activity of NKCC1 and a depolarizing shift of *E*_GABA_ in DRG neurons have been observed after nerve injury in several studies^13^,^14^,^29^,^31^. However, the net effect of the depolarizing shift of *E*_GABA_ remains unclear. It has been suggested that the shifted *E*_GABA_ might produce an enhanced PAD, which can increase excitability of primary nociceptors (hyperalgesia) or even become sufficient enough to directly evoke an action potential, thus transforming an inhibitory process into an excitatory one (tactile allodynia)^12^. Here we found that in parallel with the shift of *E*_GABA_, there is a reduction of *G*_GABA_, together leading to a loss of presynaptic inhibition, but not a switch to excitation, accounting for thermal hypersensitivity but not static mechanical hypersensitivity symptoms after nerve injury. Indeed, when we specifically knocked out the presynaptic inhibition in nociceptors, the *sns- β3*^−*/*−^ mice developed normal static mechanical hypersensitivity but no thermal hypersensitivity after nerve injury. Surprisingly, we found that the changes in *E*_GABA_ and *G*_GABA_ in DRG neurons are rather transient and not maintained at later stages, while persistent neuropathic pain symptoms are still present. Administration of bumetanide achieved anti-hyperalgesic effect in mice 2 days or 14 days after nerve injury but not in mice 21 days after injury, indicating that this transient modulation of presynaptic inhibition is mostly required for the initiation of hypersensitivity, but does not relate to the maintenance of pain in which increased excitability of projecting central areas may play a key role. This observation is strongly in agreement with a NKCC1 transient upregulation after nerve injury reported by Módol *et al.*^31^

Postsynaptic disinhibition regulated by BDNF through KCC2, thus unmasking the low-threshold input onto nociceptive-specific spinal projection neurons, has been considered as a major mechanism underlying neuropathic pain conditions, such as mechanical allodynia^2^,^6^,^15^,^47^,^48^. In primary sensory neurons, TrkB expression was primarily observed in large diameter neurons. However, BDNF has been shown to be essentially required for the postnatal survival of nociceptors and TrkB expression has also been detected in some small and medium-size neurons^49^,^50^,^51^,^52^,^53^. Ultrastructural evidence from Salio *et al.*^22^ showed that TrkB receptor is expressed at axonal terminals of primary afferent fibres in lamina II where most nociceptive fibres terminate. Furthermore, BDNF has been reported to act on these presynaptic TrkB receptors in lamina II and lead to an increase of the frequency of glutamatergic excitatory postsynaptic potentials in spinal dorsal horn of complete Freund's adjuvant-treated rats^21^,^54^. Here we report that BDNF binding to these presynaptic TrkB receptors produce both a transient depolarizing shift of *E*_GABA_ and a reduction of *G*_GABA_ in primary sensory neurons after nerve injury, leading to a transient presynaptic disinhibition and consequently to the development of thermal hypersensitivity, while postsynaptic disinhibition regulated by BDNF initiates static mechanical hypesensitivity. Our data reveals a novel functional role of BDNF in regulating presynaptic inhibition after nerve injury and open new possibilities for developing more precise and prompt therapeutic strategies for neuropathic pain treatment.

## Methods

### Animals

For all experiments, adult male mice (aged 8–10 weeks) were used. All mice were housed under a 12-h:12-h light/dark cycle. All animal experiments were done according to the German Animal Protection Law.

### Generation of *Advillin- GCaMP3* and *sns- β3* ^−*/*−^ mice

Sensory neuron-specific *Advillin- GCaMP3* mice were generated by crossing advillin-Cre^33^ transgenic mice with floxed *GCaMP3* mice^32^. Nociceptor-specific *sns- β3*^−*/*−^ mice were generated by crossing sns-cre transgenic mice^34^ with floxed β3 mice (*β3*^*fl/fl*^; JacksonLabs#008310 (ref. 55)). In all experiments, adult male littermates were strictly used to control for genetic effects of the background. Mouse genotype was verified by PCR using primers as follows: Gabrb3 Forward: 5′-ATTCGCCTGAGACCCGACT-3′; Reverse: 5′-GTTCATCCCCACGCAGAC-3′. SNS Forward: 5′-GAAAGCAGCCATGTCCAATTTACTGACCGTAC-3′; Reverse: 5′-GCGCGCCTGAAGATATAGAAGA-3′. Advillin Forward: 5′- GCACTGATTTCGACCAGGTT-3′; Reverse: 5′-GAGTCATCCTTAGCGCCGTA-3′. GCaMP3 Forward: 5′-CTT CAA GAT CCG CCA CAA CAT CG-3′; Reverse: 5′-TTG AAG AAG ATG GTG CGC TCC TG-3′.

### Real-time quantitative PCR

Total RNA was extracted from DRGs using peqGOLD TriFast (peqlab) following the manufacturer’s protocol. RNA was determined with a peqlab NanoDrop ND-1000. Complementary DNA was synthesized from 5 μg of total RNA using the SuperScript TM II Reverse Transcriptase kit (Invitrogen, Carlsbad, CA, USA) with oligo(dT) primers, according to the manufacturer’s protocol. One microlitre of 1:5 dilution cDNA was used in quantitative reverse transcriptase–PCR experiments using ABsolute QPCR SYBR Green ROX Mix (Thermo Scientific) on an ABI 7500 Real Time PCR System (ABS/Life Technologies). Primers used were as follows: Gabrb3 Forward: 5′-GCCAGCATCGACATGGTTTC-3′; Reverse: 5′-GCGGATCATGCGGTTTTTCA-3′. GAPDH Forward: 5′-ACCCTGTTGCTGTAGCCGTATCA-3; Reverse: 5′-TCAACAGCAACTCCCACTCTCCA-3.

### Induction of CCI and nociceptive tests

In deeply anaesthetized mice (isoflurane), four loose silk ligatures (4/0) were placed (with ~0.5 mm spacing) around the sciatic nerve at the level of the right mid-thigh. Ligatures were tied until they elicited a brief twitch in the respective hind limb^56^. Dynamic mechanical and thermal hypersensitivity were assessed by dynamic plantar and Hargreaves apparatus (Ugo Basile, Italy)^9^,^56^. Static mechanical hypersentivity was measured using von Frey filaments(Stoelting Europe, Ireland)^17^. The withdrawal threshold was measured for each mouse before taking DRG for culture. All experiments were performed blind to the genotype of the mice.

### Cold plantar test

The recording of cold-induced withdrawal latency was modified from previous report^57^: animals were habituated on metal platform with open grid of square holes (5 × 5 mm). Dry ice powder was packed into a 10-ml syringe and compressed into a flattened, dense pellet. The hind paws of mice were targeted by the tip of modified syringe and the withdrawal latency was measured with a stopwatch.

### DRG neurons culture

L4 and L5 DRGs were dissected and collected in a 1.0-ml tube of PBS on ice. Ganglia were cleaned, enzymatically treated and mechanically dispersed^58^. The isolated neurons were seeded on poly-L-lysine and laminin-coated coverslips. Electrophysiology experiments began 12 h after plating.

### Electrophysiology

Recordings were made from DRG neurons using fire-polished glass electrodes with a resistance of 3–7 MΩ. Extracellular solution contained the following (mM) 150 NaCl, 1 MgCl_2_, 2 CaCl_2_, 5 KCl, 10 glucose and 10 HEPES (pH 7.4), and the internal solution for filled electrodes contained the following (mM): 140 CsCl or 140KCl, 5 EGTA and 10 HEPES (pH 7.3). Membrane current and voltage were amplified and acquired using EPC-10 amplifier sampled at 10 kHz. For the perforated-patch recording, 0.1% Lucifer Yellow and 50–100 μg ml^−1^ gramicidin were included in the internal solution^24^,^58^. The formation of gramicidin perforation were monitored by the HEKA Patchmaster programme and the measurement was started until serious resistance dropped to <40 MΩ. GABA (1 mM)-induced currents were done at various holding potentials (−60, −40, −20, 0 and 20 mV) gradually. Data were analysed with Fitmaster software (HEKA) Electronik GmbH, Germany) and the reversal potential of GABA-induced current (*E*_GABA_) was calculated and fitted from the GABA-induced current amplitudes at a series of holding potentials.

### Immunmofluorescence

The DRG and spinal cords have been isolated and fixed in 2% paraformaldehyde in PBS pH 7.4 overnight and then in 25% sucrose-hanks in PBS overnight, cryo-sectioned at 10 μm and mounted on SuperFrost*/plus microscope slides and stored at –20 °C. The sections were permeablized 3 min with 0.1% Triton X-100 (Sigma-Aldrich, St Louis, MO, USA) in PBS, blocked 30 min in 1% BSA (Sigma) in PBS and incubated overnight at 4 °C with primary antibody anti-NKCC1 diluted 1:500 (goat, Santa Cruz Biotechnology, Dallas, TX, USA), anti-GABA_A_Ralpha1 diluted 1:500 (Guinea pig, Synaptic Systems, Göttingen, Germany) or anti-calcitonin gene-related peptide diluted 1:400 (Rabbit, Merck-Millipore). On the sequent day, the sections were washed in PBS and incubated 1 h at room temperature with anti-guinea pig AlexaFluor488-conjugated secondary antibody diluted 1:500 (Life Technologies, Carlsbad, CA, USA) and anti-rabbit Cy3-conjugated secondary antibody diluted 1:1,500 (Jackson Immunoresearch, West Grove, PA, USA), then washed again and mounted with ‘VectashieldTM Mounting Medium with DAPI’ (Vector Labs, Burlingame, CA, USA). The sections were viewed using an Olympus BX61 microscope equipped with epi-fluorescence illumination. Images were acquired using an Olympus XM10 charge-coupled device monochrome camera and analysed with cellSens Dimension software (OSIS). To increase spatial resolution, slices were imaged over a distance of ~10 μm within an image-stack along the *z* axis (*z*-stack, stacks distance: 0.49 μM) followed by three-dimensional deconvolution using cellSens Dimension built-in algorithm. The acquired images were analysed using the free software ImageJ (NIH, Bethesda, MD; USA.). To analyse the fluorescence intensity of the GABA_A_R alpha 1 staining, the background was reduced in every picture with the rolling ball algorithm and therefore the red, green and blue channel have been separated. The red staining (on the red channel) has been used to determine the ROI. The ROI was applied on the green channel and the green staining was analysed using the Analyze Particles built-in plugin of Image J.

### Western blotting

Proteins were isolated and pooled from six DRGs (L4 and L5 from three mice), homogenized with pestle after freeze and thaw in CelLytic MT Mammalian Tissue Lysis/Extraction Reagent (Sigma-Aldrich). Next, SDS–PAGE and western blotting were performed using the ‘XCell II SureLock Mini-Cell and XCell II Blot Module’ (Invitrogen), according to the manufacturer’s instructions. Thirty micrograms of proteins were loaded for each lane on a 4–12% Tris-glycine gel (Invitrogen), resolved and transferred onto polyvinylidene difluoride membrane. On blocking 1 h at room temperature with 5% milk powder (AppliChem, Darmstadt, Germany) diluted in double distillate water, the blotted proteins were incubated overnight at 4 °C with goat polyclonal anti-NKCC1 antibody diluted 1: 300 (Santa Cruz Biotechnology) and mouse monoclonal anti-GAPDH antibody diluted 1:10,000 (Abcam, Cambridge, UK). Next, the membrane was incubated 2 h at room temperature with the secondary antibodies ECL peroxidase-labelled anti-goat or mouse antibody (1:2,000; Amersham Biosciences, Freiburg, Germany). Labelled proteins were detected by chemiluminescence using the ECL Prime Western Blotting Detection Reagents (Amersham Biosciences) on X-ray films (AGFA, Mortsel, Belgium). The X-ray films were scanned as a grayscale picture and the bands intensity were analysed using the open source software ImageJ and calculated as expression relative to the housekeeping gene (*GAPDH*). Every lane was analysed with the plot analysis tool of ImageJ and the area of the peak corresponding to the band was used as intensity value.

### Calcium imaging

Briefly, fluorescence microscopy was done on an Observer A1 inverted microscope (Zeiss, Germany) using a 25 × 0.8 numerical aperture water immersion objective and a 175 W Xenon lamp as a light source. Before imaging, DRG neurons were incubated with 4 μM Fura-2 at 37 °C for 30–40 min and washed with extracellular solution at 37 °C for another 30 min. Excitation light was passed either through a 340-BP 30 filter or a 387-BP 16 filter. Two filters were switched by an ultra-high speed wavelength switcher Lambda DG-4 (Sutter, Novato, CA). Emissions elicited from both excitation wavelengths were passed through a 510-BP 90 filter and collected by a charge-coupled device camera (Zeiss). Different solutions were applied by multi barrel perfusion system (WAS02, DITEL, Prague). AxioVision software (Zeiss) was used to record image data. After background subtraction in each channel (*B*_340_, *B*_380_), the ratio (*R*) of fluorescence elicited by two excitation light, *B*_340_ and *B*_380_, was calculated: 
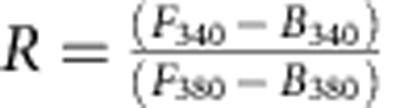
59. Data weres analysed by using AxioVision and Matlab (MathWorks, Natick, Massachusetts).

### Two-photon chloride imaging

Transgenic mice expressing Clomeleon, a ratiometric Cl^−^ indicator, under the control of the Thy1 promoter were used^60^. L4 and L5 DRG were removed and stored in oxygenized Ringer solution (124 mM NaCl, 1.25 mM NaH_2_PO_4_, 26 mM NaHCO_3_, 3 mM KCl, 2 mM MgCl_2_, 2 mM CaCl_2_, 10 mM glucose) at room temperature. A commercial 2p setup (purchased from LaVision, Germany) consisting of an upright microscope (Olympus BX51WI) and imaging software ImSpector Pro (LaVision, Germany) was used for ratiometric Cl^−^ imaging. A 20 × 1.0 numerical aperture water immersion lens (Plan-APOCHROMAT, Zeiss) was used for the experiment. Fluorescence was elicited using a Ti: Sapphire laser (Mai Tai HP DeepSee, Spectra-physics, Mountain View, CA) tuned to 870 nm. CFP signals (filter: 480 BP 36) and YFP signals (filter: 537 BP 42) were separated by a beamsplitter (500 LP) and recorded simultaneously. Time series of images or *z*-stacks were acquired from Clomeleon-positive cells in whole DRG. The data were analysed offline with ImageJ and Matlab.

### Spinal cord slice preparation

*Advillin-GCaMP3* mice that express the fluorescent calcium indicator protein, GCaMP3, exclusively in sensory neurons were used for presynaptic imaging in spinal cord slice. Control, injury and BDNF group animals were anaesthetized with ketamin:xylazine:saline (1.5:0.75:7.75). The lumbosacral spinal cord innervated by L4 and L5 spinal nerve was isolated. After removal of pia mater, the spinal cord was placed in cold Prep-Ringer solution (2–4 °C; 87 mM NaCl, 1.25 mM NaH_2_PO_4_, 25 mM NaHCO_3_, 2.5 mM KCl, 7 mM MgCl_2_, 0.5 mM CaCl_2_, 25 mM glucose, 75 mM sucrose). Next, it was embedded in 2% low melting agarose (Bio-Rad Laboratoried, CA 94547). A 300-μm transverse slice was cut from the embedded spinal cord on a vibrating blade microtome (Leica VT1200, Leica, Germany). The spinal cord slice was stored on a cellulose nitrate filter (Sartorius Stedim Biotech GmbH, Germany) perfused with Ringer solution saturated with 95% O_2_ and 5% CO_2_ at room temperature following 45–60 min perfusion at 33–34 °C for slice recovery.

### Calcium imaging with *Advillin-GCaMP3* using 2p excitation

Two-photon imaging setup is same as for chloride imaging experiments, except the laser was tuned to 920 nm. GCaMP3 signal (filter: 562 BP 40) was reflected by a beamsplitter (560 LP) and recorded. Time series of images were acquired from the spinal cord dorsal horn. Superficial layer of dorsal horn (not deeper than 100 μm from the spinal cord dorsal outline) was imaged. Scanning frequency was 1 Hz. Spinal cord slice was transferred to recording chamber of the 2p setup and perfused with oxygenized (95% O_2_, 5% CO_2_) Ringer solution. A high KCl Ringer solution was used to stimulate neurons. It contains 87 mM NaCl, 1.25 mM NaH_2_PO_4_, 26 mM NaHCO_3_, 40 mM KCl, 2 mM MgCl_2_, 2 mM CaCl_2_, 10 mM glucose. To avoid the effect of activation of glutamate receptor, 10 μM NMDA (*N*-methyl-D-aspartate) antagonist (RS)-CPP (Tocris Bioscience, UK) and 10 μM AMPA (α-amino-3-hydroxy-5-methyl-4-isoxazolepropionic acid) antagonist CNQX (abcamBiochemicals, UK) were added into high KCl Ringer solution. Ringer solution with 1 mM GABA was used to investigate neuron response to GABA. Short time perfusion with Ringer solution with GABA followed by high KCl solution with CPP, CNQX and GABA was used to investigate GABA’s influence on neuron excitation.

### Analysis of imaging data

GCaMP3 responses were analysed offline with ImageJ and Matlab. Owing to the variance of the time period required for diffusion of the applied chemicals, each recording trial was split into individual pieces according to chemical stimulations in ImageJ for later analysis in Matlab. Each piece contains 300 image frames. In each analysis, the whole recording field was segmented into 5 × 5 μm square-shaped ROIs and the same ROI settings were applied to different chemical stimulation recording pieces from the same trial. The average pixel value (*F*) in each ROI was calculated. The baseline fluorescence intensity, *F*_0_, was measured as the average of *F* during the first 30 image frames in each recording piece. The rest 270 frames were used for response measurement. When the max *F*, *F*_max_, during response frames was bigger than 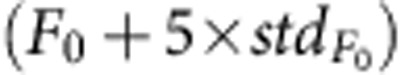, a ROI was considered to have a positive response. 
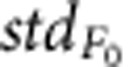
 is the s.d. of *F*_0_. *F*_max_ was converted to Δ*F* using the formula Δ*F*=*F*_max_−*F*_0_. In a given ROI, GABA is considered to have inhibitory effect on high KCl-induced response when 
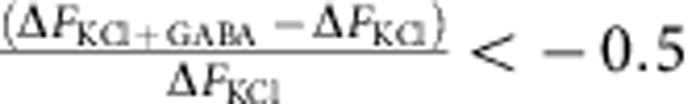
. When 
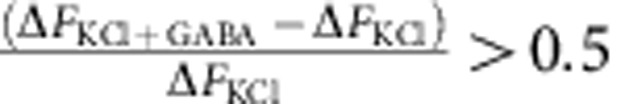
 or 
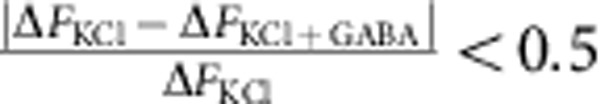
, GABA effect is considered to be excitatory or no effect, respectively. In GABA-alone stimulation piece, only ROIs showing positive responses in KCl stimulation piece from the same trial were used for analysis.

### Intrathecal injection

The mice were anaesthetized under 3% of isoflurance for induction and 1–2% for maintenance. Intrathecal injection was performed using a winged infusion set connected to a 50-μl Hamilton syringe. BDNF (50 ng kg^−1^, in 5 μl 0.9% NaCl) was injected into the intervertebral space of lumbar region between L5 and L6 level of the spinal cord. A reflexive flick of the tail was considered as a sign of the accuracy of each injection^40^. Behavioural testing was assessed by Hargreaves apparatus as above.

### Statistics

Statistical analysis was done using the Graphpad prism (GraphPad Software Inc., San Diego, CA) suite of programmes. The sample size was justified by significance testing, taking into account available number of neurons per *in vitro* experiment or mice from same litters. All means are expressed as mean±s.e.m.

## Authors contributions

J.T.-C. performed electrophysiological. J.T.-C., F.F. and F.M. did behavioural experiments. D.G. carried out calcium imaging and two-photon imaging experiments with J.T.-c.C. D.C. performed immunofluorescence and molecular biological experiments with M.K. L.Z. carried out real-time PCR. R.K. provided the sns-cre transgenic mice. P.A.H. provided the advillin-cre transgenic mice. J.H. planned experimental studies and wrote the paper.

## Additional information

**How to cite this article:** Chen, J. T.-c. *et al.* Presynaptic GABAergic inhibition regulated by BDNF contributes to neuropathic pain induction. *Nat. Commun.* 5:5331 doi: 10.1038/ncomms6331 (2014).

## Supplementary Material

Supplementary Figures Supplementary Figures 1-4.

Supplementary Movie 1 Example of time frame images in spinal cord slice from Advillin-GCaMP3 mouse without injury. Slice was challenged by high KCl, GABA and KCl+GABA in sequential.

Supplementary Movie 2 Example of time frame images in spinal cord slice from Advillin-GCaMP3 mouse 2 days after CCI injury. Slice was challenged by high KCl, GABA and KCl+GABA in sequential.

Supplementary Movie 3 Example of time frame images in spinal cord slice from Advillin-GCaMP3 mouse after BDNF treatment. Slice was challenged by high KCl, GABA and KCl+GABA in sequential.
