# Supplementary material for: Presynaptic GABAergic inhibition regulated by BDNF contributes to neuropathic pain induction
Source: Nat Commun. 2014 Oct 30;5:5331. doi: 10.1038/ncomms6331 (PMC4220496; doi:10.1038/ncomms6331)
Supplement: Supplementary Figures — 1-4. [file ncomms6331-s1.pdf]

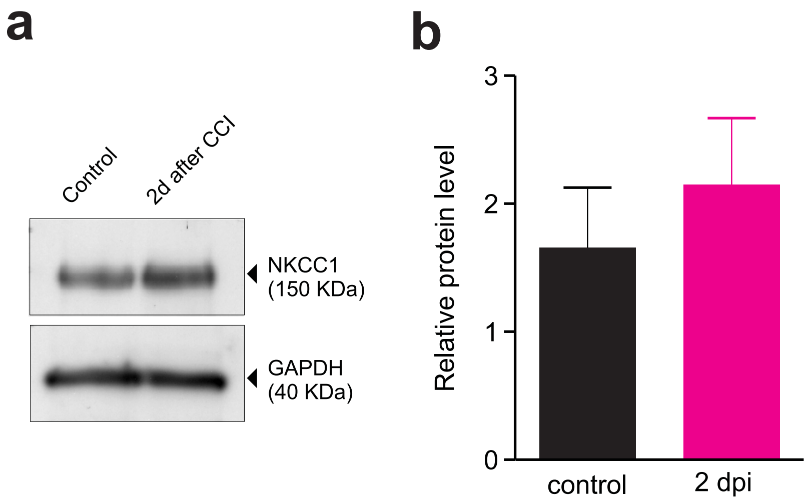

Supplementary Figure 1

Western analysis of NKCC1 expression in DRG (**a**) Western blot showing the specific band of NKCC1 at 150 KDa in DRG from control and 2 dpi animals. GAPDH at 40 KDa was used as housekeeping gene. (**b**) Histogram representing the average of the bands intensities relative to GAPDH expression in controls and 2 day after CCI. 2 samples for control and 2 samples for injury were collected. Each sample consists of 6 DRG (L4, L5) from 3 animals. 3 western blots were performed for each sample. Error bars indicate s.e.m.

Supplementary Figure-1 Hu

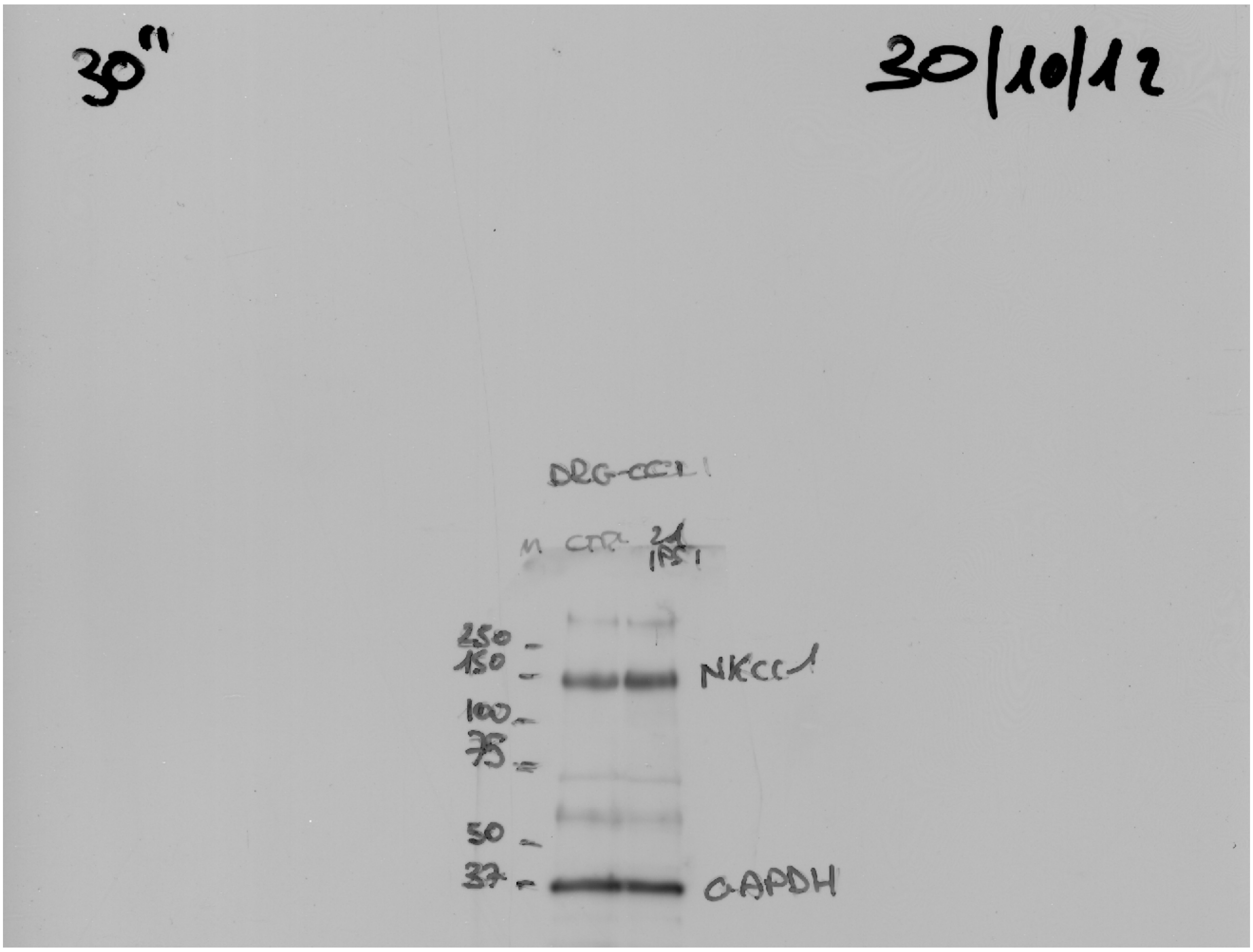

Supplementary Figure 2

Original blot presented in Supplementary Figure 1a.

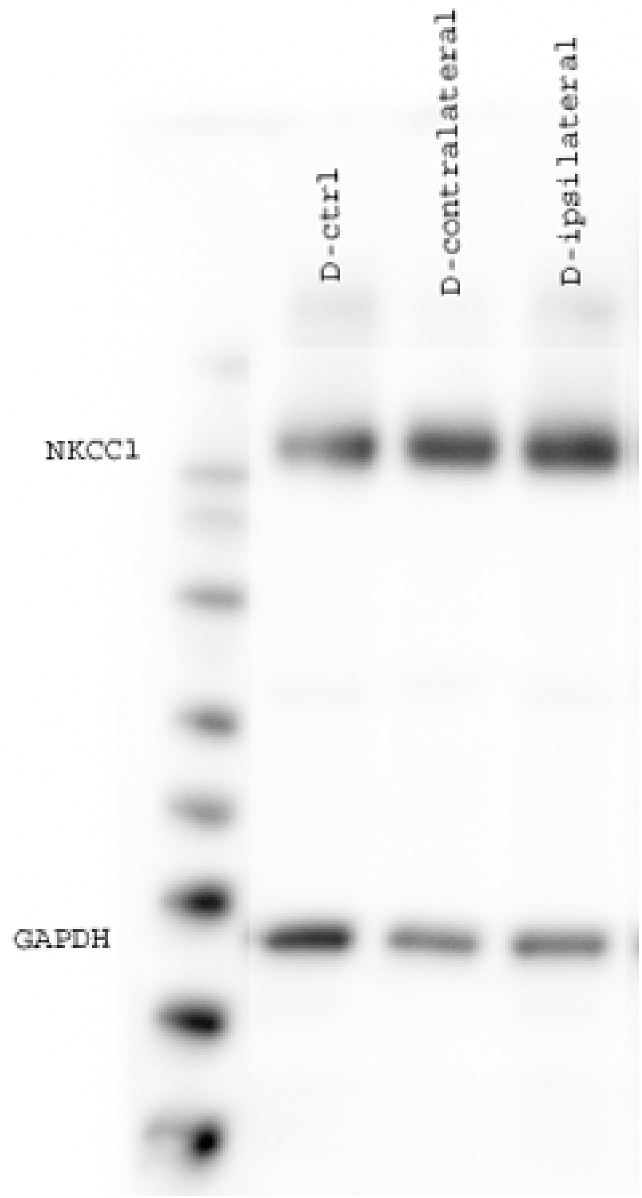

Supplementary Figure 3

Original blot presented in Figure 3h.

Supplementary Figure-3 Hu

DRG

spinal cord

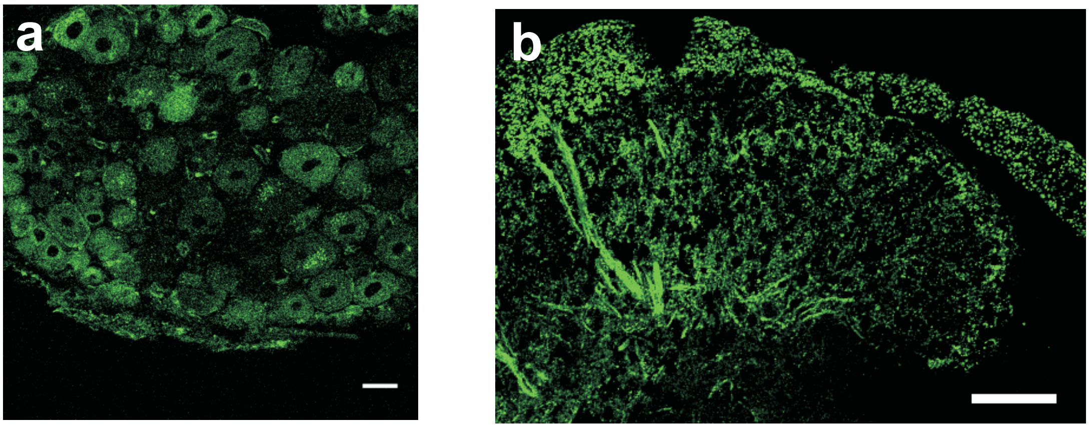

Supplementary Figure 4

mGCaMP3 expression in somata (a) and primary afferent terminals in spinal cord dorsal horn (b) of Advillin- GCaMP3 adult mouse. Scale bars: (**a**), 30μm; (**b**), 100μm.

Supplementary Figure-4 Hu

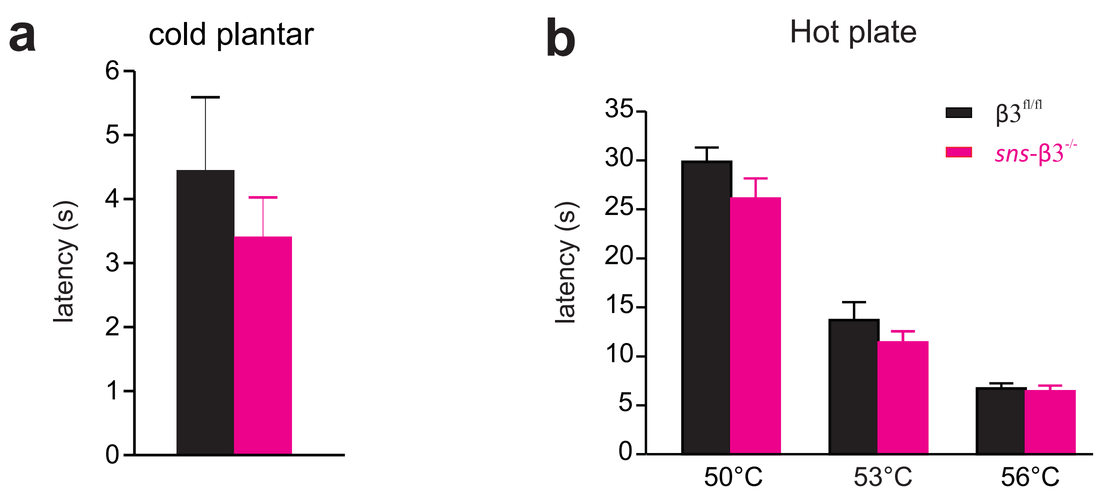

Supplementary Figure 5

Pain behavior in nociceptor specific presynaptic GABA$_A$R KO mice and littermate controls. (a) Cold plantar: *sns- β3*$^{-/-}$ mice exhibit the same sensitivity to noxious cold stimulation (*β3*$^{fl/fl}$: n=5; *sns- β3*$^{-/-}$: n=5. P>0.05). (**b**) Hot plate: no differences were observed at either 50°C, 53°C or 56°C (*β3*$^{fl/fl}$: n=11; *sns- β3*$^{-/-}$: n=12. P>0.05 for all three tested temperatures).Error bars indicate s.e.m.

Supplementary Figure-5 Hu
